# Focusing on exosomes to overcome the existing bottlenecks of CAR-T cell therapy

**DOI:** 10.1186/s41232-024-00358-x

**Published:** 2024-11-04

**Authors:** Si-Heng Zhang, Ling-Long Peng, Yi-Fei Chen, Yan Xu, Vahid Moradi

**Affiliations:** 1 Faculty of Medicine, Macau University of Science and Technology, Taipa, Macao SAR 999078 China; 2 Zhejiang Provincial Center for Disease Control and Prevention, Hangzhou, Zhejiang 310000 China; 3 Wuhu Hospital, East China Normal University (The Second People’s Hospital of Wuhu), Wuhu, 241000 China; 4 Hematology and Bood Transfusion Science Department, School of Allied Medical Sciences, Tehran University of Medical Sciences, Tehran, Iran

**Keywords:** CAR-T cell, Exosomes, Cancer immunotherapy, Targeted therapies, Anti-tumor agents, Extracellular vesicles

## Abstract

Since chimeric antigen receptor T (CAR-T) cells were introduced three decades ago, the treatment using these cells has led to outstanding outcomes, and at the moment, CAR-T cell therapy is a well-established mainstay for treating CD19 + malignancies and multiple myeloma. Despite the astonishing results of CAR-T cell therapy in B-cell-derived malignancies, several bottlenecks must be overcome to promote its safety and efficacy and broaden its applicability. These bottlenecks include cumbersome production process, safety concerns of viral vectors, poor efficacy in treating solid tumors, life-threatening side effects, and dysfunctionality of infused CAR-T cells over time. Exosomes are nano-sized vesicles that are secreted by all living cells and play an essential role in cellular crosstalk by bridging between cells. In this review, we discuss how the existing bottlenecks of CAR-T cell therapy can be overcome by focusing on exosomes. First, we delve into the effect of tumor-derived exosomes on the CAR-T cell function and discuss how inhibiting their secretion can enhance the efficacy of CAR-T cell therapy. Afterward, the application of exosomes to the manufacturing of CAR-T cells in a non-viral approach is discussed. We also review the latest advancements in ex vivo activation and cultivation of CAR-T cells using exosomes, as well as the potential of engineered exosomes to in vivo induction or boost the in vivo proliferation of CAR-T cells. Finally, we discuss how CAR-engineered exosomes can be used as a versatile tool for the direct killing of tumor cells or delivering intended therapeutic payloads in a targeted manner.

## Introduction

Almost all living cells—prokaryotes and eukaryotes—produce and secrete nano-scale membrane-bound vesicles known as extracellular vesicles [1]. Based on the biogenesis mechanism, extracellular vesicles are divided into two main classes: ectosomes and exosomes. Ectosomes are cellular products with diameters ranging from 50 nm to 1 μm that are directly derived from the plasma membrane through a mechanism called outward budding [2]. Invagination of the endosomal membrane through inward budding results in the production of multivesicular bodies that contain intraluminal vesicles. Finally, the intraluminal vesicles are secreted to the extracellular regions as exosomes (with a diameter of 40 to 160 nm) through the fusion of multivesicular bodies to the plasma membrane [2, 3].

Due to the cross-paths between multivesicular bodies with the intracellular vesicles and organelles, they carry several bio-molecules of their parental cells, including nuclear and cytosolic proteins, metabolites, DNA, different types of RNA (mRNAs and non-coding RNAs), lipids, cell surface markers, and other biologic pieces of information [4]. While in the past it was assumed that exosomes are used by cells to dispose of redundant or unnecessary cellular contents, today it has become clear that exosomes are not only a garbage bag and play an essential role in cellular communications and biological processes [4]. By loading their contents inside exosomes and secreting them, different cells can affect and regulate the function of other cells in distant areas. For example, malignant cells produce a high number of exosomes, which play pivotal roles in tumor progression, metastasis, angiogenesis, and immunoregulation [5]. Or immune cell-derived exosomes can act instead of their cellular counterparts in areas far from the reach of cells [6, 7]. In addition, the biocompatibility, natural cellular entry, and low immunogenicity and toxicity of exosomes suggest them as potential tools for delivering therapeutic payloads [8]. Exosomes can also be genetically or metabolically engineered to deliver their contents in a targeted manner to desired tissues and cells [9]. In recent years, by growing knowledge about exosomes, they have emerged as efficient diagnostic and prognostic factors, therapeutic targets, and therapeutic tools [10].

Despite the outstanding results of CAR-T cell therapy, several limitations limit its therapeutic efficacy and widespread use. Thus, in recent years, several attempts have been focused on the optimization of CAR-T cell therapy to enhance its safety and effectiveness. These efforts include the optimization of CAR design, developing non-viral approaches to CAR-T cell manufacturing, lessening CAR-T-related toxicities, and improving the durability and anti-tumor function of CAR-T cells [11, 12]. In recent years, the growing knowledge about exosomes has highlighted their undeniable role in facilitating cellular crosstalk so that it has become evident that they can affect the fate of any cellular-based therapy, such as CAR-T cell therapy [13].

**Fig. 1:**
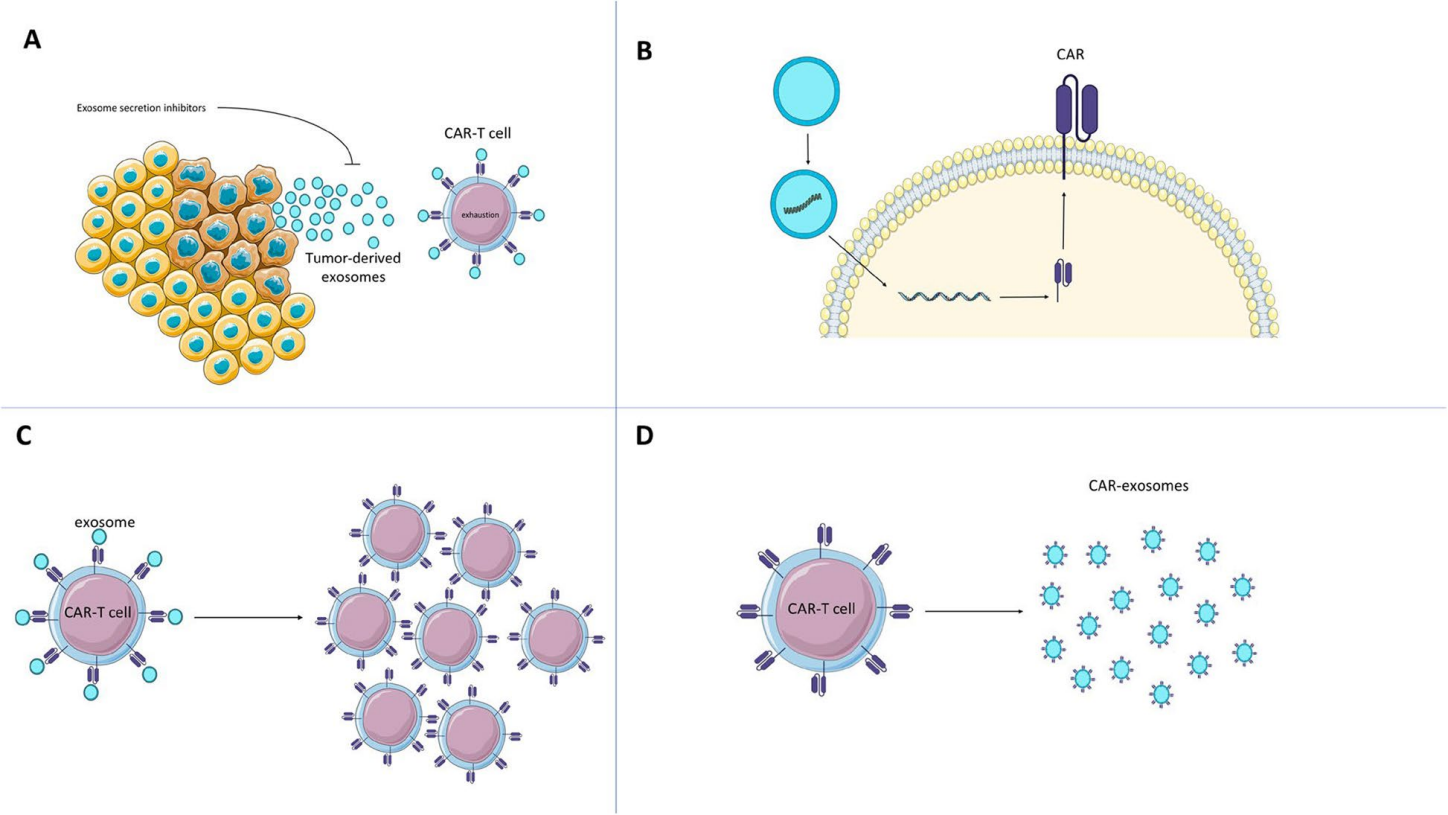
Focusing on exosomes to address the existing bottlenecks of CAR-T cell therapy. **A** Inhibiting the secretion of tumor-derived exosomes can improve the persistence and function of CAR-T cells. **B** Exosomes offer an innovative, non-viral method for manufacturing CAR-T cells, both ex vivo and in vivo. **C** Engineered exosomes can be utilized for ex vivo activation and expansion of CAR-T cells or as an adjuvant therapy to promote the in vivo proliferation of CAR-T cells. **D** Cell-free CAR-based immunotherapy using CAR-exosomes can overcome several limitations of their cellular counterparts. CAR-exosomes are capable of exerting direct cytotoxic activity against cancer cells and can also be used as targeted delivery vehicles for anti-cancer agents

In this comprehensive review, we highlight the potential for overcoming the current bottlenecks of CAR-T cell therapy by leveraging the unique properties of exosomes. In the initial sections, following a brief overview of CAR-T cell therapy and its limitations, we delve into the effect of tumor-derived exosomes on CAR-T cell function and discuss how inhibiting their secretion can enhance the efficacy and therapeutic window of CAR-T cell therapy. Afterward, we discuss how exosomes can be applied as gene delivery vehicles to produce CAR-T cells in a fully non-viral approach and remove the safety concerns and exorbitant cost of current virus-based approaches. Moreover, we discuss how this method can be translated into in vivo production of CAR-T cells. We also explore the potential of engineered exosomes in ex vivo activation and expansion of CAR-T cells and compare this method with currently available commercial kits. The application of engineered exosomes in boosting the in vivo proliferation of CAR-T cells has also been covered. Finally, we discuss how CAR-engineered exosomes can be applied as a versatile tool for the direct killing of cancer cells or delivering intended therapeutic payloads in a targeted manner (Fig. 1).

## An overview of CAR-T cell therapy

T lymphocytes, specifically CD8 + cytotoxic T lymphocytes, are the most important immune cells in fighting against cancers; nonetheless, malignant cells can avoid being recognized by host T cells using various mechanisms. For example, the downregulation of major histocompatibility complex (MHC) molecules in cancer cells can render them invisible to T lymphocytes, as the target cell recognition by T cells depends on the presentation of antigens by MHC-I/II molecules [14]. During the years 1989 to 1993, two scientists from Israel named “Gideon Gross” and “Zelig Eshhar” designed a chimeric T cell receptor that enabled T cells to recognize target cells without relying on antigen presentation by MHC molecules. They showed that by linking the variable region of the heavy and light chain of an antibody to the intracellular part of the T cell receptor (TCR), a chimeric receptor is composed that has the same specificity as the used immunoglobulin and can engage with target antigens and trigger T cell responses. Today, this construct is referred to as first-generation CARs. T cells engineered by first-generation CARs were innovative and showed promising results in vitro but lacked favorable in vivo activity [15–17].

In 1998, an important step was taken in optimizing the CAR structure. Sadelain et al. added CD28 to the CAR structure, which significantly increased the in vivo durability and function of CAR-T cells [18]. Currently, we know that costimulatory molecules other than CD28, such as 4-1BB and OX40, can be used in the CAR constructs. Today, CARs with a single costimulatory domain are called second-generation [19]. In the years that followed, more optimizations were made in the CAR structure, which led to the introduction of the third-, fourth-, and fifth-generation CARs. Third-generation CARs benefit from signals of two costimulatory domains. Although the third-generation CARs were innovative, they showed no enhanced efficacy compared to the previous generation. Fourth-generation CARs, also called TRUCKs (“T cells redirected for universal cytokine-mediated killing”) or armored CAR-T cells, use an additional transgene to secrete immunomodulatory cytokines (e.g., IL-2) upon CAR signaling [20]. In this regard, a nuclear factor of activated T cell (NFAT)-responsive cassette, which contains a transgenic cytokine, is transferred into T cells. Thus, the transgene is induced to express when the CAR binds to its targeted antigen and triggers signal transduction. Fifth-generation CARs—also called next-generation—utilize various approaches to boost the efficacy and safety of CAR-T cells. The most promising approach in this generation is based on the use of a truncated intracellular domain of a cytokine receptor (e.g., IL-2R) with a motif for binding and activation of transcription factors such as JAK/STAT pathways [21]. Another promising approach in this generation is the use of switch receptors that lead to the introduction of more controllable CAR-T cells. ON-switch or OFF-switch receptors enable scientists to control the activation, apoptosis, or depletion of CAR-T cells upon administration of an exogenous agent [22]. Thanks to the significant advancement brought about by the introduction of second-generation CARs, 11 approved CAR-T products are commercially available (Table 1).

**Table 1:** Commercially available CAR-T cells

Generic name	Trade name	Developer	Target Ag	Delivery strategy	CAR structure		Approved for	Date of approval	Approving authority	Cost per infusion	Ref
					**Hinge/transmembrane**	**Costimulatory domains**					
Tisagenlecleucel	Kymriah	Novartis	CD19	LVV	CD8α/CD8α	4-1BB + CD3ζ	Pediatrics and young adult B cell acute lymphoblastic leukemia	2017	FDA	$475,000	[23]
							Adults large B cell lymphoma	2018			
Axicabtagene ciloleucel	Yescarta	Gilead Sciences	CD19	RVV	CD8α/CD8α	CD28 + CD3ζ	Large B-cell lymphoma	2017	FDA	$473,000	[24]
							Follicular lymphoma	2021			
Brexucabtagene autoleucel	Tecartus	Gilead Sciences	CD19	RVV	CD28/CD28	CD28 + CD3ζ	Mantle cell lymphoma	2020	FDA	$373,000	[25]
							B cell acute lymphoblastic leukemia	2021			
Lisocabtagene maraleucel	Breyanzi	Bristol-Myers Squibb	CD19	LVV	IgG4/CD28	4-1BB + CD3ζ	Large B cell lymphoma	2021	FDA	$410,300	[26]
Idecabtagene vicleucel	Abecma	Bristol-Myers Squibb	BCMA	LVV	CD8α/CD8α	4-1BB + CD3ζ	Multiple myeloma	2021	FDA	$419,500	[27]
Ciltacabtagene autoleucel	Carvykti	Janssen Biotech, Inc	BCMA	LVV	CD8α/CD8α	4-1BB + CD3ζ	Multiple myeloma	2022	FDA	$465,000	[28]
Relmacabtagene autoleucel	Carteyva	JW Therapeutics	CD19	LVV	CD8α/CD8α	4-1BB + CD3ζ	Follicular lymphoma	2021	NMPA		[29]
Varnimcabtagene Autoleucel	ARI-0001	Hospital Clinic	CD19	LVV	CD8α/CD8α	4-1BB + CD3ζ	B cell acute lymphoblastic leukemia	2021	AEMPS		[30]
Inaticabtagene autoleucel	CNCT 19	Juventas	CD19			4-1BB + CD3ζ	B cell acute lymphoblastic leukemia	2023	NMPA		[31]
Equecabtagene autoleucel	Fucaso	IASO Biotechnology	BCMA	LVV	CD8α/CD8α	4-1BB + CD3ζ	Multiple myeloma	2023	NMPA		[32]
Zevorcabtagene autoleucel	Zevor-Cel, CT053	CARsgen Therapeutics	BCMA	LVV	CD8α/CD8α	4-1BB + CD3ζ	Multiple myeloma	2024	NMPA		[33, 34]

*FDA* Food and Drug Administration, *NMPA* China National Medical Products Administration, *AEMPS* The Spanish Agency of Medicines and Medical Devices, *LVV* lentiviral vector, *RVV* gamma-retroviral vector, *BCMA* B cell maturation antigen

## Existing bottlenecks of CAR-T cell therapy

The currently approved CAR-T products are manufactured through an individualized process in which patients’ own lymphocytes are isolated by leukapheresis and infused back into patients after ex vivo engineering to express CAR. The customized manufacturing approach makes it an expensive and time-consuming treatment [35, 36]. While many patients cannot afford this treatment due to its exorbitant cost, there is also the risk of production failure for other patients. It is related to the immunosuppressive mechanisms of tumor cells and anti-cancer drugs, which render patients’ T cells unable to expand ex vivo [36, 37]*.* The manufacturing process of autologous CAR-T cells usually takes around 2–5 weeks. Since this treatment is only used for patients with end-stage or progressive disease, this delay in manufacturing is so long that patients may pass away during this period or no longer be eligible for this treatment [38]. In recent years, there has been a surge in interest in developing a universal, off-the-shelf product. As the use of allogeneic CAR-T cells is hindered by the risk of graft versus host disease (GvHD) and their immunogenicity, in recent years, several approaches have been developed to expand the applicability of allogeneic CAR-T cells. These strategies include the use of programmable nucleases or small hairpin RNAs (shRNA) to knockout or downregulate TCR and MHC molecules to prevent GvHD and immune rejection of allogeneic CAR-T cells. Another approach is the use of effector cells with a lower risk of GvHD, including T cell subsets with limited TCR diversity and effector immune cells that lack the TCR expression [38]. Nonetheless, all the clinical trials of allogeneic CAR-T cells are still in the initial phases and have not yet been approved by regulatory authorities.

Despite the outstanding outcomes of CAR-T cell therapy in B-cell derived malignancies, this therapy has not lived up to expectations in solid tumors due to the tumor heterogenicity and suppressive role of the tumor microenvironment (TME). The choice of an antigenic target is the first challenge in CAR-T cell therapy of solid tumors since the tumor-associated antigens are also expressed by normal cells, which increases the risk of on-target off-tumor toxicity [39]. On the other hand, the expression of tumor-associated antigens is not consistent between the malignant cells, which makes it difficult to eradicate all the malignant cells with a single specific CAR-T product [40]. Moreover, CAR-T cell infiltration into tumor sites is impeded by various physical barriers that limit the access of CAR-T cells to tumor cells [41]. Additionally, the immunosuppressive circumstance of TMEs impairs the proliferation and cytotoxic function of CAR-T cells. TMEs have several immunosuppressive cells (such as myeloid-derived suppressor cells and regulatory T cells) and soluble factors (such as tumor growth factor β and interleukin-10) that inhibit CAR-T cells that have successfully entered the tumor site [41]. Moreover, the abundance of ligands of T cell immune checkpoint inhibitors such as PD-L1 on the malignant cells renders CAR-T cells exhausted and non-functional [42].

There is also the risk of on-target on-tumor toxicities, which are related to the engagement of CAR-T cells with malignant cells and their excessive responses. For example, excessive release of cytokines such as IL-1, IL-6, and INF-γ can lead to systemic inflammation referred to as cytokine release syndrome (CRS). It can be mild, moderate, or severely fatal [43]. Tumor lysis syndrome is another possible complication after CAR-T cell therapy, which occurs as a result of the sudden or massive destruction of malignant cells by CAR-T cells and the release of their contents [44]. Immune effector cell-associated neurotoxicity syndrome (ICANS) is a neurologic side effect of CAR-T cell therapy, which may occur days or weeks after treatment. Although the exact cause of ICANS is not completely grasped, it appears that endothelial activation-induced blood–brain barrier (BBB) breakdown due to the release of inflammatory cytokines is the cause of neurotoxicity [45]. Also, rare cases of severe life-threatening macrophage activation syndrome (MAS)/hemophagocytic lymphohistiocytosis (HLH) have been reported after CAR-T cell therapy [46]. All the mentioned items underscore the need to develop a method to lessen CAR-T cell-related toxicities.

## Inhibiting the secretion of tumor-derived exosomes to improve the function of CAR-T cells

Like other cells, cancerous cells secrete extracellular vesicles, which act as cell-free contributors in cell–cell communications [47]. Tumor-derived exosomes (TEXs) are also called “oncosomes” because they have an important role in tumorigenesis, tumor progression, metastasis, angiogenesis, immune evasion, and induction of drug resistance [48]. TEXs bear several biomolecules of their parental cells, such as lipids, proteins, and nucleic acids, and can regulate the function of target cells by transmitting these contents [49]. Exosomes are mirrors of their origin cells. Most of the changes that have occurred in malignant cells are also seen in their derived exosomes. These changes include changes in the expression level/pattern of non-coding RNAs, metabolites, proteins, and other biological contents. One of the other differences between malignant and normal cells is that the exosome secretion activity of malignant cells is much higher than normal cells. It has been revealed that cancer cells produce more exosomes than normal proliferating cells. It has also been shown that in patients with cancers, the number of exosomes in their body fluids is significantly more than that of healthy individuals [48, 49]. The body fluids of cancer patients pose a broad group of extracellular vesicles that are derived from malignant and non-malignant cells. Although the role of exosomes may vary depending on tumor types and other conditions, several reports indicate the facilitating role of TEXs in tumor growth, metastasis, and immune evasion [49]. For example, TEXs can spread to distant organs prior to tumor cells reaching and forming a pre-metastatic niche that is hospitable for metastatic malignant cells [48]. Studies have revealed that under hypoxic conditions, the malignant cells secrete more exosomes, which can modulate the tumor extracellular matrix and facilitate angiogenesis or metastasis of tumor cells to a more suitable area [50].

In the context of anti-cancer immune responses, the TEXs can be considered a double-edged sword. While several studies indicate the immunosuppressive function of TEXs, there is some evidence that TEXs encourage the anti-cancer function of immune cells. For example, it has been shown that TEXs containing heat shock protein 70 (HSP70) and/or Bcl-2–associated athanogene 4 (Bag4) can trigger migration of natural killer (NK) cells to tumor sites and elicit their cytolytic activity or IFN-γ production [51–54]. TEXs may carry tumor-associated antigens and deliver them to antigen-presenting cells (APCs), leading to the prime of T cell activation and anti-tumor responses [55]. On the other hand, numerous studies have shown that TEXs have an immunosuppressive effect on host immune cells. It appears that while TEXs activate immune cells at early stages, sustained activation and overstimulation of immune cells by TEXs puts them in an exhausted state and suppresses their antitumor response [52, 56].

The immunosuppressive role of TEXs is not limited to NK cells, and TEXs can directly or indirectly reprogram or inhibit the function of a broad spectrum of immune cells. TEXs pose several membrane receptors and ligands that originate from their parent cells and enable them to interact with immune cells. Additionally, they can be internalized by immune cells and deliver their contents (such as micro RNAs) into the recipient cells [49]. TEXs may exert different effects on CD8 + T cells and CD4 + T cells. Wieckowski et al. have revealed that TEXs inhibit the signaling and proliferation of CD8 + T cells and induce apoptosis in them while not exerting a negative effect on CD4 + T cells. They also demonstrated that TEXs increase in vitro expansion and suppressive function of regulatory T (Treg) cells [57]. It has been demonstrated that exosomally expressed TGF-β and NKG2D ligands suppress the expression of NKG2D receptors on CD8 + T and NK cells and impair their NKG2D-mediated function [58–61]. Tumor-derived exosomes also reprogram the peripheral blood monocytes, which prevents them from differentiation into functional dendritic cells and skews them towards myeloid-derived suppressor cells (MDSCs) [62]. TEXs can reprogram M1 macrophages to polarize toward the M2 phenotype and enhance the pro-tumor function of M2 macrophages [63].

**Fig. 2:**
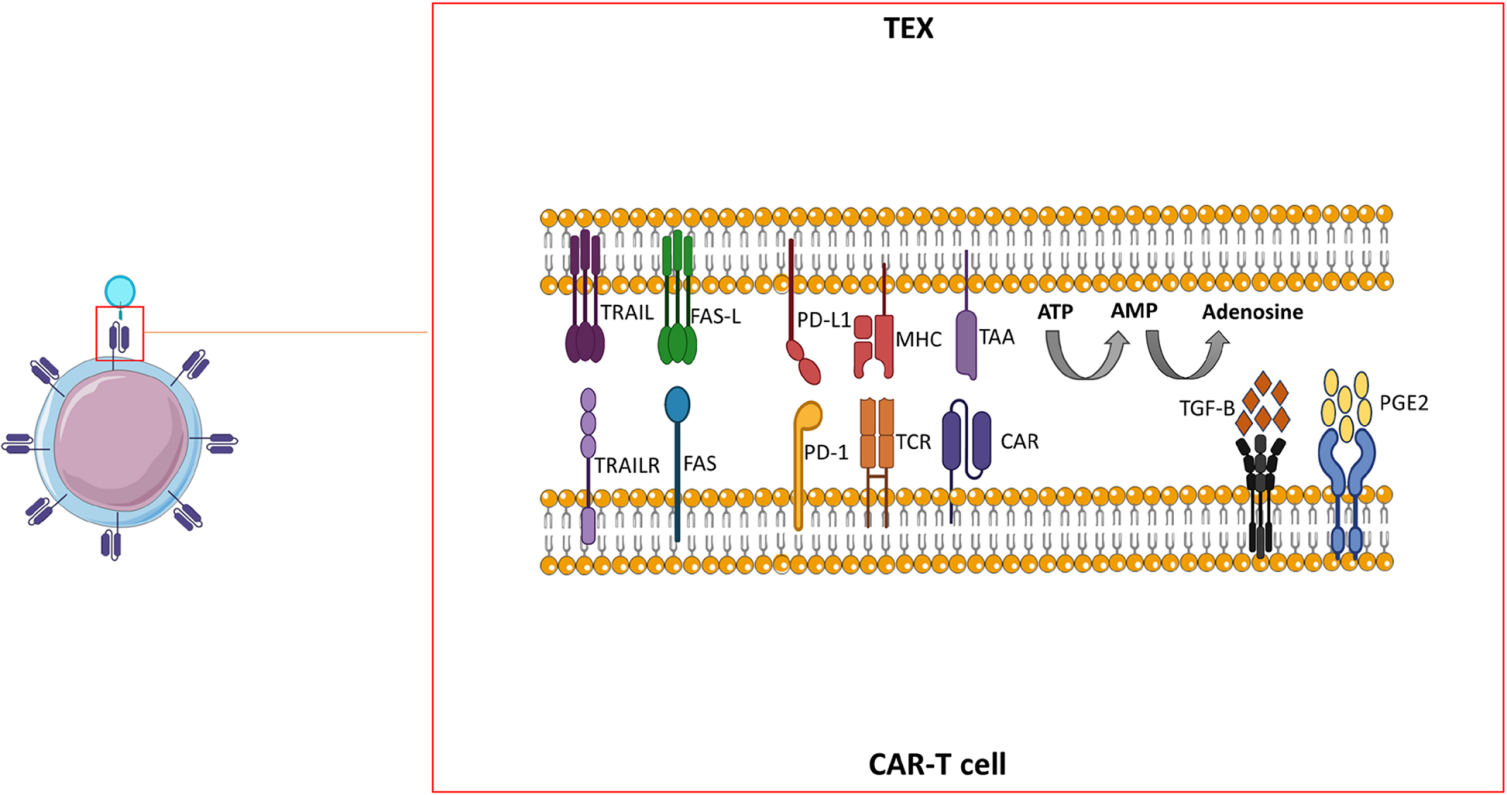
Interaction between CAR-T cells and tumor-derived exosomes. Tumor-derived exosomes are able to impair the function of CAR-T cells before they reach tumor sites by various mechanisms, including overstimulation of CAR-T cells, downregulation of CAR-T cell activity by immune checkpoint molecules, secretion of inhibitory factors such as TGF-β and PGE2, and converting ATP and AMP molecules to adenosine

There is evidence that TEXs can negatively affect patients' response to chemotherapy and immunotherapy [48]. It is estimated that after infusion of CAR-T cells, up to 10 million extracellular vesicles per CAR-T cell can exist in the peripheral blood of recipients, implying a high risk of CAR-T cell dysfunction by extracellular vesicles [64]. TEXs carry both tumor-associated antigens and ligands of immune checkpoint inhibitors (such as PD-L1) on their outer membrane. They can suppress the proliferation, migration, and function of CAR-T cells (Fig. 2) [65]. In a study that has been conducted by Cox et al., it has been observed that TEXs of patients with chronic lymphocytic leukemia (CLL) have a higher level of PD-L1 on their outer membrane, which can induce a state of exhaustion in CAR-T cells. Nonetheless, this effect was not reversed by PD-L1 blocking antibodies, suggesting that in addition to PD-1-PDL-1 interaction, other mechanisms are also involved in this regard (46). CD73 and CD39 molecules on the membrane of TEXs can increase the level of adenosine through dephosphorylation of ATP and 5′AMP. High levels of adenosine have a detrimental impact on CAR-T cells and can impair their proliferation [66]. Another possible underlying mechanism of CAR-T cell dysfunction is attributed to chronic stimulation of CAR by TEXs. TEXs carry tumor-associated antigens that can specifically bind to the CAR through antigen–antibody interaction and induce constant stimulation in CAR-T cells. This long-term exposure leads to terminal differentiation of CAR-T cells and overexpression of exhaustion markers (CTLA-4, LAG3, TIM3, and PD1), senescence markers (such as CD57), and T cell inhibitory receptors (such as TGIT and tumor necrosis factor receptor superfamily) [67]. It has been revealed that CD19 + lymphoma-derived exosomes activate CD19 CAR-T cells at early stages, but at later stages, CAR-T cells enter a state of exhaustion due to the constant signaling of CAR. Additionally, it has been shown that lymphoma-derived exosomes skew CAR-T cells toward differentiated phenotypes or regulatory T (Treg) cells [68]. Zhu et al. showed that the activation of CAR-T cells by TEXs stimulates them to invalid production and secretion of proinflammatory cytokines and enters them in an exhausted state [69]. In a study by Ali et al., it has been observed that neuroblastoma-derived exosomes impair the anti-tumor function of CD4 + antiCD171 CAR-T cells but not CD8 + CAR-T cells. The authors infer that depending on the original tumor type and CAR-T cell subsets, the immunosuppressive effects of TEXs may differ [70].

Given the substantial role of TEXs in the development of cancers and their resistance to treatment, in recent years, several drugs to prevent their production and secretion have been developed. Sphingomyelinase inhibitors, RAB27A-targeting agents, and pharmacological inhibitors are the three main classes of TEX inhibitors that can prevent the secretion of TEXs without affecting the secretion of soluble proteins [71]. In mouse models, it has been revealed that blocking the secretion of TEXs can enhance tumor infiltration and anti-tumor function of both CAR-T and endogenous immune cells. [65]. Zhang et al. have revealed that overexpression of Rab27a as a result of upregulation of hypoxia-inducible factor-1α (HIF-1α) promotes the production of CD19 + exosomes in CD19 + B-cell malignancies. They showed that anti-Rab27a small interfering RNA (siRNA) can prevent the secretion of TEXs and encourage responses to chemotherapy [72]. By combining CAR-T cell therapy with TGF-β inhibitors, the suppressive effects of TEXs on CAR-T cells can be effectively counteracted [68]. Finally, the level of circulating TEXs can be diminished by therapeutic plasma exchange or dialysis before CAR-T cell therapy [73].

## Engineered exosomes as non-viral CAR delivery tools

All CAR-T products approved by regulatory authorities worldwide have been produced using integrated lentiviral or gamma-retroviral vectors (Table 1). Although CAR-T cells produced using lentiviral/retroviral vectors have shown promising outcomes, clinical use of integrating viral vectors faces several challenges. Production of lentiviral/retroviral vectors is a time-consuming process that includes the design of viral packaging plasmids, packaging cell line expansion, packaging cell line transfection, viral soup harvesting, viral soup concentration and purification, sterilization, and titration of viral vectors. This process may take several months [74].

Additionally, to minimize the risk of safety concerns about the clinical application of lentiviral/retroviral vectors, several requirements that regulatory authorities have established must be met. Production of clinical-grade lentiviral/retroviral vectors under current good manufacturing practice (cGMP) requires high cost, high-tech facilities, and specialized personnel, which only a few laboratories around the world can match [74–76]. As a result, in recent years, several attempts have been made to develop a safe, efficient, and cost-beneficial non-viral vector. Various non-viral vectors have been developed in recent years that can yield permanent or transient expression of CAR, including transposon vectors, gene editing tools, mRNA, minicircles, and nanoplasmids [12]. These vectors can be produced at a lower cost and in a shorter time than viral vectors, and their use also addresses most of the safety issues about lentiviral/retroviral vector-mediated gene transfer. On the other hand, one of the distinct advantages of viral vectors over non-viral vectors is their natural cell entry, which yields a high transduction rate. Nevertheless, non-viral vectors cannot penetrate cells, and an additional delivery technique is required to transfer them into the target cells [77]. Several physical and chemical delivery strategies have been introduced during the past years, among which electroporation is the most popular delivery technique. In this technique, cells are exposed to high-voltage electrical pulses, which transiently permeabilize them for exogenous agents. Nonetheless, the use of electroporation comes with several irreversible side effects, such as the death of a significant percent of electroporated cells, alteration of gene expression level, and reduction of viability and expansion of CAR-T cells [78]. In recent years, nanoparticles have emerged as an efficient mRNA delivery tool into cells. The use of nanoparticles, specifically lipid nanoparticles, for CAR mRNA delivery into the T cells has shown favorable results in preclinical studies [12, 79]. Nonetheless, the use of nanoparticles is associated with some limitations, such as low biocompatibility and low degradability. In addition, mRNA-loaded nanoparticles have poor stability and must be kept at ultra-low temperatures, which makes them difficult to transport and handle [80–82].

Exosomes have several characteristics that render them potential gene delivery vehicles, including biocompatibility, cell tropism, viral-like transfection efficiency, high cargo capacity, high stability, low clearance rate, and low immunogenicity [83–85]. Moreover, exosomes can be engineered to bind to the intended cells and tissues ex vivo or in vivo [86]. Exogenous cargo can be loaded into exosomes by various loading methods including incubation methods, transfection methods, physical methods, and in situ assembly and synthesis. In incubation methods, the exogenous cargo can be incubated with exosomes or exosome-secreting cells, which results in the packaging of the desired cargo in exosomes. In the transfection strategy, three different methods can be used: (i) transfection of the exosome-secreting cells to overexpression of intended cargo, which increases their packaging within exosomes; (ii) direct transfection of exosomes, or (iii) transfection of exosome-secreting cells with desired cargos that are fused to specific chaperons for enhancing their loading into exosomes. Physical methods facilitate the loading of exogenous agents into exosomes by membrane recombination processes (these methods include extrusion, dialysis, and freeze–thaw) or by induction of transient pores on the exosomal plasma membrane (these methods include electroporation, sonication, and surfactant treatment) [87].

In a recently published study, Si et al. developed engineered exosomes with the dual ability to activate T cells and deliver CAR mRNAs to them. Their designed exosomes express anti-CD3/CD28 single chain variable fragments (scFvs) on their surface, which activate T cells by engaging with their membrane CD3/CD28 and facilitate delivery of CAR mRNA into T cells. To enhance the wrapping of intended mRNAs within the exosomes, they utilize the exosomal loading capability of lysosome-associated membrane protein 2 isoform B (LAMP-2B), which is an exosomal marker. In this strategy, the bacteriophage coat protein MS2 is linked to the C-terminus of LAMP-2B, and an MS2 binding site (MS2bs) is inserted into the 3’ untranslated region (UTR) of desired mRNA (in this case CAR). Therefore, the MS2 interacts with MS2bs in the 3 UTR of CAR mRNAs and enhances their loading into secreted exosomes [88]. It has been revealed that by using cargo-loading chaperones such as LAMP-2B and VSVG, the exosomal packaging of intended mRNAs can increase by 6 to 40 times [87]. Through the use of modified exosomes developed by Si et al., T cells were successfully activated, and CAR expression was induced in them in ex vivo experiments [88]. In the coming years, this innovative method can pave the way for in vivo induction of CAR-T cells and circumvent the current drawbacks of ex vivo manufacturing. However, more modifications and optimizations are required to ensure that exosome-mediated CAR-T cells have a killing efficacy on par with CAR-T cells manufactured by viral vectors [88].

Clinical translation of natural cell-derived exosomes is faced with the limitation of large-scale production. On the other hand, natural cell-derived exosomes are highly heterogeneous, which makes their quality control process difficult. Accordingly, in recent years, research into the generation of artificial exosomes has gained significant momentum. Artificial exosomes are generally produced by three main methods: (i) top-down strategies, (ii) bottom-up strategies, and (iii) biohybrid strategies [89]. To the best of our search, the artificial exosomes have not yet been used for CAR-T cell production; however, it is expected that by further progress in manufacturing, purification, and loading of artificial exosomes, they will be tested in the field of CAR-T cell therapy.

## In vivo*/*ex vivo activation and expansion of CAR-T cells using engineered exosomes

### Ex vivoactivation and expansion

To reach a clinically meaningful number of CAR-T cells, it is crucial to use an optimal strategy for the activation and expansion of T cells. Currently, the protocols mostly rely on cross-linking monoclonal antibodies (anti-CD3 and anti-CD28) in combination with specific cytokines such as interleukin (IL)-2 [90]. These monoclonal antibodies are usually coated on magnetic beads, polymer beads, or nanotube scaffolds and are added to T cell culture media [91, 92]. As the transduction rate of T cells by viral or non-viral methods is not 100%, the non-specific techniques lead to the activation and expansion of both transduced and non-transduced T cells. Therefore, additional purification by magnetic-activated cell sorting (MACS) or fluorescence-activated cell sorting (FACS) methods may be required for the purification of CAR-T cells. Artificial antigen-presenting cells (aAPC) can provide an antigen-specific activation method. These aAPCs, by expressing tumor-associated antigens and providing multiple co-stimulations as well as lacking the expression of MHC molecules, lead to specific activation of CAR-expressing cells [93, 94]. Nevertheless, the safety concerns around the use of living cells and the necessity of removal of the aAPCs by additional processes are the main drawbacks of these methods [95, 96].

In recent years, it has been shown that exosomes can be engineered to express desired receptors/ligands on their outer surface, enabling them to bind to a specific cell type. In this regard, engineered exosomes can be used to activate and expand T cells in an antigen-specific or non-specific manner. As described previously, Si et al. developed an engineered HEK 293 T cell-derived exosome for the activation of T cells and the production of CAR-T cells. They revealed that anti- “CD3/CD28 scFvs”-expressing exosomes efficiently engage with T cells and induce their activation. There were no differences between CD3/CD28-expressing exosomes and commercial CD3/CD28-coated magnetic beads in the expansion rate of T cells and their differentiation state. This method can promise a more affordable method for T cell activation and expansion in the future [88]. In a recent study, Ukrainskaya et al. developed a novel strategy capable of activating and expanding CAR-T cells in a specific manner while avoiding the out-growth of CAR-negative T cells. They utilized Hella cell-derived membrane vesicles with the expression of CAR target antigen on their outer membrane. For increasing membrane blebbing, they treated Hella cells with cytochalasin B [94]. Cytochalasin B is a cell-permeable mycotoxin that blocks actin polymerization and enhances cell extrusion and formation of extracellular vesicles with the same membrane molecules as parental cells [97, 98]. They showed that the generated extracellular vesicles only activate the cognate CAR-T cells but not co-existing non-transduced T cells. The engineered extracellular vesicles showed comparable and, in some cases, better efficiency in activation and expansion of CAR-T cells than conventional methods. This strategy led to fivefold more expansion of CAR-T cells while reducing the required time for expansion. EV-expanded CAR-T cells had a higher proportion of less differentiated T cells as well as a lower number of exhausted cells than conventionally generated CAR-T cells, which is a good prognosis of treatment efficacy [94]. The authors hypothesize that these results are due to the difference in the specific stimulation signals via CAR engagement with non-specific stimulation via CD3/CD28 engagement. In sum, this strategy offers an optimal and scalable method for the ex vivo expansion of CAR-T cells. Hella cells or other cell lines can be engineered to express CAR-target antigens and be used for a long time to generate extracellular vesicles. In addition, extracellular vesicles could be stored to be used for multiple patients [94]. Engineered exosomes can yield a meaningful number of CAR-T cells with a higher purity in a shorter time while preventing their terminal differentiation and exhaustion. Moreover, as discussed in the previous section, engineered exosomes can be used as a single modality for transferring CAR transgene into T cells and activating T cells simultaneously [88]. Through subsequent validation and optimizations, this method can be used instead of both viral-mediated engineering and microbeads-mediated activation of T cells. Additionally, this method has great potential for in vivo induction of CAR-T cells. Thus, finally, it should be noted that this section compares the exosome-mediated activation and expansion of CAR-T cells with the traditional method. However, this is an emerging method that is performed in a few proof-of-concept pre-clinical studies and requires further validation and optimizations.

### Engineered exosomes to enhance in vivo proliferation of CAR-T cells

In vivo proliferation and durability of functional CAR-T cells are crucial for the efficacy of treatment. Various studies indicate a clear correlation between the high percentage of less mature T cells in the final CAR-T product and enhanced clinical responses. Naive (TN), central memory (TCM), and stem-like memory (TSCM) T cells are self-renewable and more proliferable, leading to the more durability of CAR-T cells and long-term remission of patients [99]. Accordingly, multiple strategies have been developed in recent years to sustain the less-differentiated stage of T cells during CAR-T cell manufacturing. Nonetheless, in an antigen-sparse microenvironment, there may not be enough stimulations for CAR-T cell expansion [100]. Accordingly, in recent years, different strategies have been used to enhance the in vivo proliferation of CAR-T cells by providing antigenic stimulation. For example, in mouse models, the use of RNA and peptide vaccines led to modifying dendritic cells to present CAR target antigen to CAR-T cells and stimulate their proliferation, which was associated with better anti-tumor responses [101–103]. In a phase I clinical trial (NCT03186118), patient-derived T cell antigen-presenting cells (T-APCs) were transduced to express truncated human CD19 antigen and infused into patients’ post-CAR-T cell therapy, which led to long-term remission of treated patients [104]. The results of clinical trials that have been published so far indicate the safety of the clinical use of exosomes [105]; thus, engineered exosomes with CAR ligands on their membrane could be administered in vivo to stimulate CAR-T cells. Using this method, the need for antigen presentation by antigen-presenting cells (APCs) is bypassed. In a recently published study, Zhang et al. produced CAR ligand-engineered exosomes to induce in vivo proliferation of CAR-T cells. For this purpose, they transduced HEK-293 T cells with the *CD19* transgene and infused the HEK 293 T-derived CD19 + exosomes into mice post-CAR-T cell therapy [106]. Published results demonstrate the safety of HEK 293 T-derived exosomes and their efficiency in promoting the proliferation and persistence of patient-derived and healthy donor-derived CAR-T cells. The authors stated that exosomes with CAR ligands significantly promote the durability of CAR-T cells and diminish the risk of disease relapses. One of the concerns around the use of this strategy is the exhaustion of CAR-T cells as a result of chronic stimulation. However, the authors state that by using a limited dose of exosomes, CAR-T cells efficiently can be expanded with minimal risk of exhaustion. Using this method, the required time for ex vivo expansion of CAR-T cells can be drastically diminished and provide a faster treatment for patients [106]. Therefore, in the coming years, with further investigations and optimizations, adjusted doses of CAR ligand-expressing exosomes can be administered as an adjuvant to CAR-T cell therapy.

## Cell-free CAR-based immunotherapy using CAR-exosomes

**Fig. 3:**
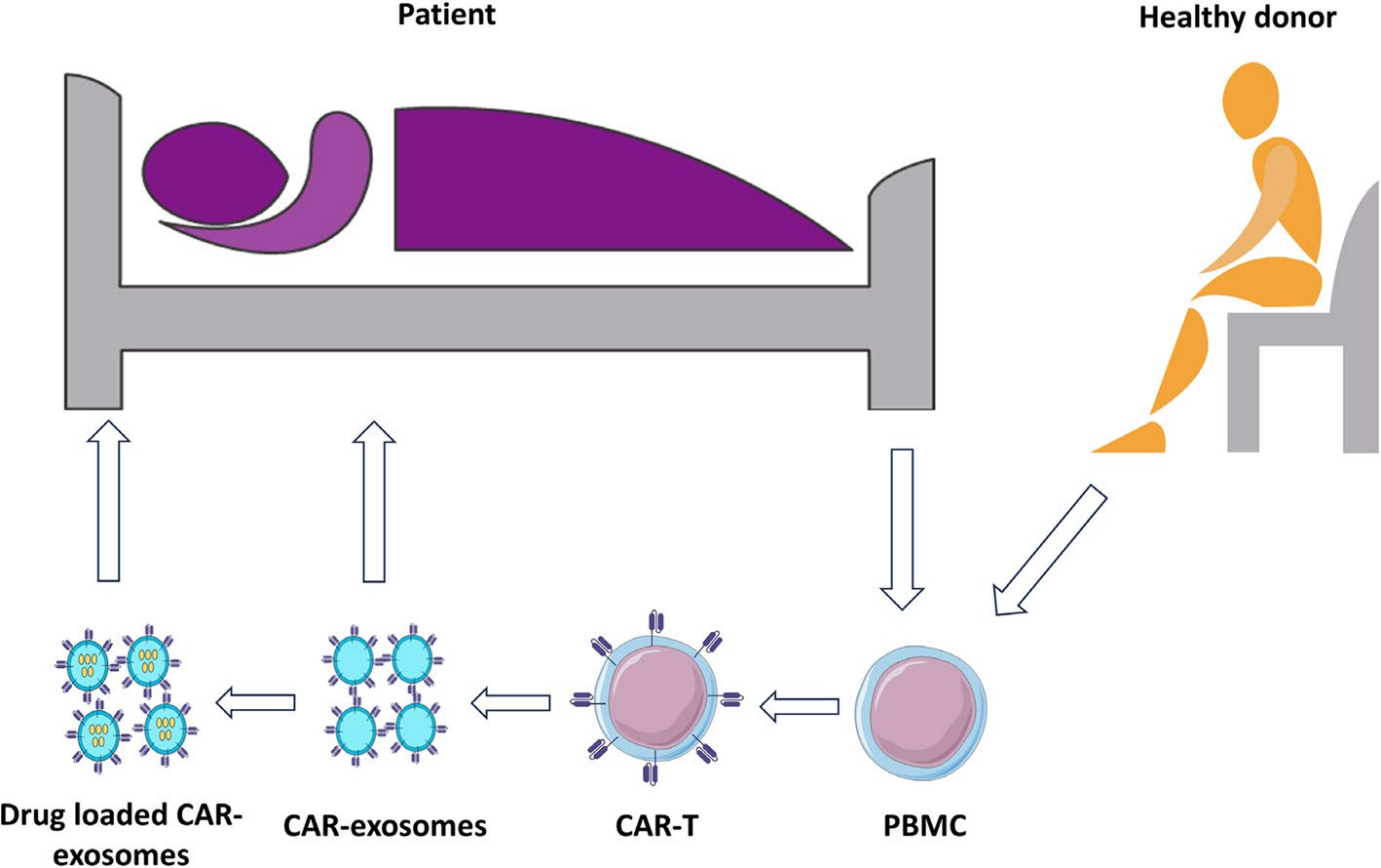
Schematic procedure of CAR-exosome therapy. CAR-exosomes can be sourced from either autologous or allogeneic settings. CAR-exosomes can be used as a direct cytotoxic agent to fight against cancer or be loaded with intended therapeutic payloads for their targeted delivery to the malignant cells

Immune cells produce several types of extracellular vesicles that can directly suppress tumor growth or promote the anti-tumor function of other immune cells (107). Nonetheless, immune cell-derived exosomes are not specific, so they cannot deliver their contents to specific cells. Engineering exosomes to express CAR on their outer surface is a potential way to redirect them toward a specific cell type. In the subsequent sections, the potential of CAR-exosomes as direct cytotoxic agents and targeted delivery vehicles (Fig. 3) is discussed.

### CAR-exosomes as direct cytotoxic agents

As mentioned before, the low efficacy of CAR-T cells in the treatment of solid and dense tumors, as well as the management of its post-treatment complications, remain leading concerns. Immune cells, like other living cells, secrete extracellular vesicles that play an essential role in anticancer immune responses by mimicking the function of their origin cells [107]. In recent years, the advent of CAR-T-derived exosomes (CAR-exosomes) has opened a new avenue for overcoming the limitations of the use of CAR-T cells, as they have several advantages over their cellular counterparts (Table 2). The nanometer diameter size of CAR-exosomes gives them the ability to penetrate physical barriers such as the blood–brain barrier or the blood–tumor barrier [4]. This can be substantiated by findings showing that TEXs can cross the tumor’s physical barriers and reach body fluids [108, 109]. Therefore, CAR-exosomes can infiltrate tumor sites more efficiently than CAR-T cells, which is a distinct advantage in the treatment of solid tumors. In addition, CAR-exosomes mirror most of the characteristics of CAR-T cells, such as killing efficiency, which makes them an emerging tool to fight against cancers. Several preclinical studies have been conducted in recent years using CAR-T-derived exosomes, and their results indicate the efficacy and safety of CAR-exosomes in the treatment of solid tumors and hematologic malignancies [110–112]. CAR-T-derived exosomes carry several cytotoxic molecules, such as perforin, FAS-ligand, Apo2L, granzyme A, and granzyme B, which originate from their parental cells. They are also negative for PD-1 expression, which makes them superior to their cellular counterparts in resisting the immunosuppressive effect of the tumor microenvironment [113, 114]. The cell-free treatment using CAR-exosomes removes the risk of leukemic transformation of CAR-T cells that have been reported in some recent cases [115].

**Table 2:** Comparison of CAR-T cells and CAR-exosomes

	CAR-T cells	CAR-T-derived exosomes
In vivo life span	Higher	Lower
Proliferation ability	Can proliferate	Cannot proliferate
Immunogenicity	Higher (specifically in allogeneic settings)	Lower
Targeted specificity	Directed via CAR	Directed via CAR
Killing mechanisms	Direct cytotoxicity, cytokine production	Direct cytotoxicity, delivery of therapeutic payloads
The risk of toxicities (CRS, ICANS, GvHD)	Higher	lower
Infiltration into tumor sites	Low infiltration into solid tumors	Better access even to condensed tumor
Across from biological barriers	Low penetrance	High penetrance
Sensitivity to PD-L1	Sensitive	Insensitive
Leukemic transformation	Has been reported	Is not possible
Storability	Storable	Storable
Re-administration	Challenging	More straightforward

Fu et al., in a preclinical study, revealed that the antitumor function of CAR-exosomes is antigen-specific and is directed via CAR. They showed that anti-EGFR CAR-exosomes and anti-Her2 CAR-exosomes efficiently kill the EGFR + and Her2 + malignant cells, respectively, while not affecting the target cells that are negative for EGFR and Her2. In all xenograft models of this study, in vivo administration of CAR-exosomes efficiently inhibits tumor growth in a dose-dependent manner. In contrast to CAR-T cells, the antitumor function of CAR-exosomes was not affected by PD-L1 both in vivo or in vitro, indicating the absence of PD-1 expression on exosomes. Finally, they showed that in vivo administration of CAR-exosomes is safer than their cellular counterparts and does not cause CRS [110]. In another preclinical study, Yang et al. demonstrated the safety and efficacy of anti-mesothelin (MSLN) CAR-exosomes in the treatment of MSLN + triple-negative breast cancer.

The study’s findings strongly suggest that CAR-exosomes exert their cytolytic activity by secreting perforin and granzyme A and B at the immunological synapse [112]. However, despite these findings, the mechanism of the anti-tumor function of CAR-exosomes has yet to be fully identified and requires further research. In an interesting study, it has been shown that CAR-exosomes derived from HEK293T cells that were transduced with anti-CD19 CAR have cytotoxicity against CD19 + cells in a CAR-dependent fashion [111]. Given the absence of T cell cytotoxic granules in HEK293T cells, these results raised questions about the underlying mechanisms of the antitumor activity of CAR-exosomes. In this study, it was observed that CD19 CAR-exosomes were taken up by both CD19 + and CD19-negative target cells but only induced cytotoxicity in CD19 + target cells. The authors infer that while CAR exosomes can be taken up by both CD19 + and CD19-negative target cells, downstream cytotoxicity signals are only initiated when binding is mediated by CAR [111]. Depending on the cell origin of exosomes, they may carry several biomolecules that are harmful or beneficial for cancer cells. For example, natural killer cell-derived exosomes containing perforin, granzyme A and B, and granulysin can kill malignant cells [116]. Therefore, engineering other immune cell-derived exosomes with CAR can also be explored in subsequent studies.

It has been shown that treatment using CAR-exosomes is safer than treatment with their cellular counterparts. Compared with conventional CAR-T cell therapy, the risk of CRS, ICANS, and GvHD is significantly lower in CAR-exosome therapy [110, 117]. Thus, they can be used in allogeneic settings and as an off-the-shelf product.

Despite encouraging results of CAR-exosomes in several preclinical studies, it should be noted that CAR-exosomes are in their start room and have a long way to go in clinical settings. For example, results from various studies have revealed that T cells secrete multiple types of exosomes with opposite functions. While some of the T cell-derived exosomes inhibit tumor growth and activate other immune cells [118, 119], some others accelerate tumor progression and suppress immune responses [120, 121]. This underscores the need for further research to characterize immune cell-derived exosomes and their contents better. Currently, the dose of exosomes is adjusted based on the weight of isolated exosomes in micrograms, and the number of CAR molecules on exosomes can be calculated using methods like ELISA [110]. Determining the maximum tolerated dose is critical to eliciting the best antitumor response while avoiding toxicity. By further optimization, CAR-exosomes can be used in combination with CAR-T cells or as an off-the-shelf product to remove the existing limitations of autologous CAR-T cell therapy.

### CAR-exosomes as targeted delivery vehicles

Since the exosomes can be taken up by malignant cells [122], CAR-exosomes can be loaded with various therapeutic agents to deliver them to malignant cells. Treatment with drug-loaded CAR-exosomes is similar to a combination therapy, where the direct cytolytic activity of CAR-T-derived exosomes and the anticancer effect of the loaded drug can synergistically reduce tumor burden [123].

In a study by Xu et al., CAR exosomes have been successfully utilized to deliver CRISPR/Cas9 components to malignant cells both in vitro and in vivo. They transduced the HEK293T cell line by anti-CD19 CAR transgene and loaded the HEK293T cell-derived CAR-exosomes with plasmids encoding MYC-targeting sgRNA/Cas9 complex. CRISPR-loaded CAR-exosomes were taken up by CD19 + malignant cells more efficiently than CRISPR-loaded unmodified exosomes and significantly inhibited tumor growth [124].

Anti-tumor agents are generally administered intravenously in clinical settings; however, exosomes have a unique biodistribution that leads to their accumulation in metabolic organs [125, 126]. Therefore, choosing a suitable route for in vivo administration of exosomes is a very crucial step. For example, it has been shown that intracardial injection of CAR-exosomes prevents their accumulation in the liver and/or spleen and enhances their infiltration into tumor sites [124]. Inhalation of drug-loaded exosomes is another emerging approach to the targeted delivery of anti-cancer drugs to the lung while avoiding systemic toxicities [127, 128]. In a recently published study, paclitaxel (a chemotherapeutic drug) was successfully encapsulated within anti-mesothelin CAR-T-derived exosomes, and the paclitaxel-loaded CAR-exosomes were administered to xenograft models by inhalation. The researchers revealed that inhaled paclitaxel (PTX)-loaded CAR-exosomes efficiently distributed to the lung and inhibited tumor growth while avoiding systemic toxicity. These engineered exosomes also led to an increased number of CD8 + T cells and elevated levels of TNF-α and IFN-γ in the tumor microenvironment [123]. In another recent study, Zhu et al. designed a hybrid nanovesicle called LipCExo@PTX, which was composed by fusing anti-mesothelin and anti-PD-L1 bi-specific CAR-T cell-derived exosomes with lung-targeted liposomes and loaded these nanovesicles by PTX. In this strategy, tissue tropism of lung-targeted liposomes is combined with the cell specificity of CAR-exosomes, leading to targeted delivery of therapeutic payloads. After intravenous injection of LipCExo@PTX, over 95% of nanovesicles accumulated in the lung. They showed that anti-mesothelin CARs facilitate the delivery of PTX and cytotoxic granules inside nanovesicles to tumor cells, and the anti-PD-L1 CARs on the surface of LipCExo@PTX efficiently reverse the immunosuppressive effect of the tumor microenvironment [129].

Lexus et al. showed that engineering CAR-T cells to deliver pattern recognition receptor agonists is an effective solution to promote the anti-tumor function of CAR-T and other endogenous immune cells. They engineered CAR-T cells to carry non-coding RNA RN7SL1, which acts as a damage-associated molecular pattern (DAMP) and activates RIG-I/MDA5 signaling in immune cells. RN7SL1 promotes the proliferation and effector-memory phenotype of CAR-T cells. Additionally, they showed that RN7SL1 is deployed in CAR-T-derived exosomes and regulates the function of endogenous immune cells. In this regard, RN7SL1 reduces the proportion of myeloid-derived suppressor cells (MDSCs) and the level of TGF-β while promoting activation and expansion of tumor-specific T cells and the immunostimulatory phenotype of dendritic cells [130].

Nonetheless, the use of CAR exosomes for targeted delivery of therapeutic cargoes faces several challenges. Most importantly, it remains difficult to package cargo within exosomes in the desired quantity. It has been reported that exosomes contain caspase 3 and may deliver it to tumor cells, leading to resistance to cell death and inhibiting the accumulation of chemotherapy agents in tumor cells. Poor pharmacokinetics of drug-loaded exosomes have also been reported [131]. Moreover, cells secrete a diverse population of exosomes, which can be different in biological function, underscoring the need for a better understanding of the heterogeneity of exosomes. As discussed above, the choice of administration root is another crucial step in exosome-based therapies [86].

### Production and quality control of CAR-exosomes

The production of exosomes begins with the cultivation of their parent cells. In this regard, T cells are activated in vitro and engineered to express CAR. While flask-based static systems can support lab-scale studies, the use of 3D bioreactor systems allows cell culture on a large scale and increases the yield of exosome production. Bioreactors are being developed to increase the exosome production yield or to prevent contamination of exosomes with the nutrients and other contaminants in the culture medium. For example, equipping bioreactors with shear stress inducers can increase the exosomes budding by cells up to 20-fold [132].

The downstream process of exosome production includes their concentration and purification. The gold standard technique for separation of exosomes from culture media is ultracentrifugation with 100,000–200,000 g force. Nonetheless, ultracentrifugation has a lower yield and also carries the risk of aggregation of exosomes and their contamination with cellular debris and macromolecules; thus, the technique is not suitable for large-scale and clinical-grade exosome generation [133]. As an alternative technique, tangential flow filtration can concentrate the exosome-rich conditioned media up to ~ 100 × . Several studies show that the yield, purity, and batch-to-batch consistency of exosomes produced by tangential flow filtration are higher compared to those produced by ultracentrifugation [134–136]. Super absorbent polymer beads are another potential technique that allows the concentration of exosomes on large scales and in compliance with cGMP [137]. After concentration, anion exchange chromatography (AIEX) or size exclusion chromatography (SEC) can be used for further purification of concentrated exosomes [138, 139].

After these processes, exosomes should be characterized based on the minimal information for studies of extracellular vesicles 2018 (MISEV2018) [140]. Quantification of exosomes can be performed by determining the total protein amount (using BCA protein assay) and total particle number (using nanoparticle tracking analysis). Protein content-based characterization of exosomes is based on analyzing at least one protein of the following categories: (i) transmembrane or GPI-anchored proteins, (ii) cytosolic proteins, and (iii) non-EV co-isolated structures such as lipoproteins, apolipoproteins, albumin, tamm-Horsfall protein, and ribosomal proteins [140]. CAR-T cell-derived exosomes can be characterized by evaluating the presence of CAR and other typical exosomal proteins and the absence of exosome-negative markers, including Golgi protein (GM130), endoplasmic reticulum protein (calnexin), nuclear protein (lamin B1), and mitochondria protein (prohibitin). The amount (ng) of CAR protein per μg CAR exosome/CAR-T cell can be measured to compare the CAR expression level between CAR exosomes and CAR-T cells. Results indicate that the CAR protein level in the same μg of CAR-T cells and CAR-exosomes is comparable [110].

While in previous studies determining the total particle number of exosomes per total protein (pn/µg) was used to assess exosome purity, size exclusion high-performance liquid chromatography (SE-HPLC) provides a more precise technique to determine the purity of the product. To reduce the risk of immunogenicity in clinical-grade exosome products, measuring the residual host cell DNA (by residual host cell DNA quantitation kit) and host cell proteins (by ELISA) outside the exosomes is crucial. Final products should also be assessed for mycoplasma, adventitious virus, endotoxins, and sterility [141].

### Bottleneck of CAR exosomes and their potential solutions

Although published preclinical results indicate the safety and efficacy of CAR exosome therapy, the affordability of treatment for patients is another determinant that should be considered for translating CAR exosomes from bench to beside. Although the potential for CAR exosomes to be developed as off-the-shelf products could make them more cost-effective than autologous CAR-T cell therapy, several other determinants may influence the final cost of this therapy.

The overall cost of CAR exosome therapy is related to the costs of cell culture, exosome isolation, concentration, purification, quality control, and storage. Other factors that influence the final price of the treatment include the required dose of the product and the number of times it is administered. Large-scale production of exosomes in compliance with cGMP is challenging and expensive [132]. On the other hand, although exosomes are storable, it has been revealed that their long-term storage can induce their aggregation and reduce their efficacy [142]. Adjusting the dose of CAR exosomes is another challenge in the clinical application of CAR exosomes. The dose of exosomes in most preclinical studies varies between 10–100 µg exosomal protein/mouse [143]. Nonetheless, due to the lack of a universal method for producing CAR exosomes, the yields of CAR exosomes produced by different groups may differ. An alternative method is based on quantifying the CAR expression using ELISA. In a study by R et al. it has revealed that 5 × 104 CAR-T cells or 10 μg CAR exosomes have comparable in vitro cytotoxicity. They also showed using ELISA that 5 × 104 CAR-T cells and 10 μg of CAR exosomes contain 10 ng and 6 ng of CAR protein, respectively [110]. Thus, it can be inferred that 10 ug CAR exosomes containing 10 ng CAR protein have a killing ability comparable to 5 × 104 CAR-T cells. Nonetheless, the cytotoxicity of specific amounts of CAR exosomes can vary depending on the production protocol, the type of the used CAR, and its target antigen. By further validation of the safety and efficacy of CAR exosomes, initial phase dose-adjusting clinical trials can be conducted to obtain the maximum tolerated dose of CAR exosomes in clinical settings. Another factor that can affect the cost and efficacy of treatment is the in vivo durability of CAR exosomes. It has been revealed that CAR-T cells generated by non-integrating mRNA vectors have limited efficacy in treating malignancies. It is attributed to the transient expression of CAR by mRNA-mediated CAR-T cells [144, 145]. In these cases, repeated administration of CAR-T cells may be required to induce complete remission; nonetheless, this increases the overall cost of treatment. Similar issues exist in CAR exosome therapy. Due to exosomes’ limited life span [146], repeated administration is critical to maintain antitumor activity, which imposes an extra cost burden on recipients.

Further optimization in the protocols for the clinical grade and large-scale generation of CAR exosomes in a reproducible manner is crucial for their clinical translation. In the following, we present suggestions that can help reduce the overall costs of CAR exosome therapy. The use of transposon vectors (such as sleeping beauty and piggyBac vectors) instead of lentiviral/retroviral vectors can significantly diminish the cost of CAR-T cell manufacturing, reducing CAR exosome therapy’s price since manufacturing clinical-grade transposon vectors is considerably simpler and cost-beneficial than clinical-grade viral vectors [74]. Another strategy to minimize CAR exosome therapy’s overall cost is using induced pluripotent stem cells (iPSCs) as the master cell source for generating CAR exosomes. IPSCs, as a potentially unlimited cell source, can support the sustainable production of CAR-T cells and their derived CAR exosomes. Nonetheless, differentiation of iPSCs into CAR-T cells on large scales and in compliance with cGMP is challenging [147]. Another strategy to reduce the cost of CAR exosome therapy involves using a split, universal, and programmable (SUPRA) CAR system. This system comprises a universal signal transduction receptor named zipCAR and a soluble antigen-binding domain called zipFv [148]. T cells or iPSCs can be engineered to express zipCAR. After the isolation, concentration, and purification of zipCAR-expressing exosomes, adding zipFv leads to binding these two parts and forming a functional CAR. Using this approach, CAR exosomes with different specificity can be generated from the same master cell source.

Using conventional methods for exosome production, about 1 µg of exosomal protein is usually obtained per milliliter of culture medium. A strategy for scaling up CAR exosome production is based on the use of stress stimuli, which increase the exosome secretion activity of cultured cells. These stimuli include serum starvation, acidifying the pH of the culture medium, thermal stress, hypoxia, specific additives in the culture medium, chemical inducers (sulfhydryl-blocking agents, Ca2 + , cytochalasins, H2O2, vesiculation buffer, hyaluronic acid, and specific drugs), and physical inducers (shear stress, irradiation, exposure to ultrasound, magnetic force, and microgravity). Nonetheless, these techniques increase the risk of contamination of the final product by chemical contaminants and cellular components such as nuclear DNA and organelles [132].

In recent years, artificial exosomes (biomimetic vesicles) have emerged as novel alternatives to natural exosomes to overcome their drawbacks, such as low yield and heterogeneity. Nanovesicles, exosome-mimetics, and hybrid exosomes are the three main types of artificial exosomes, which are prepared using top-down, bottom-up, and biohybrid strategies, respectively [149].

In the top-down approach, parental cells are manipulated to be disassembled and generate nanovesicles. Several top-down strategies have been developed in recent years, including extrusion, filtration, microfluidic devices, sonication nitrogen cavitation, and chemical-induced cell blebbing. Since the parental cells are used as the master source, nanovesicles have the highest similarity with natural exosomes among all three types of artificial exosomes. Several results indicate nanovesicles have membrane structure, size, morphology, distribution, zeta potential, and protein markers similar to natural exosomes [89]. It has been revealed that the generation of nanovesicles is 100 times faster than the same amount of natural exosomes, and the overall yield of nanovesicles is about 100-fold more than natural exosomes [150, 151]. Although nanovesicles take advantage of higher yield and faster production than natural exosomes, loss of cytoplasmic content, contamination with nuclear components or other contaminants, and the lack of sustainability due to the destruction of parental cells are the main drawbacks of nanovesicles [152].

In contrast to the top-down strategy, in the bottom-up approach, small molecules and components are combined in a stepwise process to generate exosome-mimetics. Liposomes, the most well-known exosome-mimetics, consist of phospholipid bilayers surrounding an aqueous core [153].

In the biohybrid strategies, natural exosomes are merged with synthetic nanoparticles by co-extrusion, freeze-thawing, or incubation. These strategies sum up the high stability, homogeneity, and high production yield of synthetic nanoparticles with properties of natural exosomes, including biocompatibility, low immunogenicity, and the ability to penetrate biological barriers [89]. As mentioned ago, Zhu et al. developed a hybrid exosome by merging anti-mesothelin and anti-PD-L1 bi-specific CAR exosomes with lung-targeted liposomes, which combine the tissue tropism of lung-targeted liposomes with the cell specificity of CAR-exosomes. This hybrid structure enabled the targeted delivery of PTX into the malignant lung cells of mice [129]. Although they have not yet been widely used in CAR-based immunotherapy, artificial exosomes (especially nanovesicles and biohybrid exosomes) hold great potential to overcome the current bottlenecks of CAR exosomes, including low yield, low homogeneity, limited life span, and high cost.

## Conclusion

In this review, we investigated how bottlenecks of CAR-T cell therapy can be overcome by focusing on exosomes and the processes they are involved in. Given the critical role of tumor-derived exosomes in CAR-T cell dysfunction, combining CAR-T cell therapy with exosome secretion inhibitors could be used in future clinical trials to assess whether this approach could lead to better outcomes or not. The potential of exosomes to be loaded with various molecules and engineered to express desired ligands on their outer surface makes them an emerging tool for ex vivo manufacturing of CAR-T cells by safer, time-saving, and cost-beneficial methods than current methods. Moreover, due to biocompatibility, low immunogenicity, and delivery capabilities, in the coming years, the use of exosomes can create a new era in CAR-T cell therapy by furthering the dream of in vivo induction of CAR-T cells. According to the safety of exosomes, engineered exosomes with expression of CAR target antigen can be used as an adjuvant to boost in vivo proliferation and durability of CAR-T cells. Finally, cell-free CAR-based immunotherapy using CAR-exosomes promises a safe and efficient target therapy approach that avoids several limitations of cell-based treatments.

The use of exosomes in CAR-T cell therapy is just being started, and yet there is still a long way to go. Although exosomes can be manufactured rapidly and in large amounts, their clinical-grade large-scale manufacturing needs to be optimized. It is expected that by further optimizations in the production, loading, engineering, and dosing of exosomes, more preclinical studies in the field of CAR-T cell therapy using exosomes will be conducted, and exosomes-based approaches gradually will be entered into CAR-T clinical trials.

## Data Availability

Not applicable.

## Abbreviations

CAR: Chimeric antigen receptor
CAR-T cell: Chimeric antigen receptor T cell
CAR-exosome: Chimeric antigen receptor-expressing exosomes
TEXs: Tumor-derived exosomes
EV: Extracellular vesicles
LVV: Lentiviral vector
RVV: Gamma retroviral vector
GvHD: Graft versus host disease
CRS: Cytokine release syndrome
ICANS: Immune effector cell-associated neurotoxicity syndrome
